# Developmental Toxicity of Ochratoxin A in Rat Embryo Midbrain Micromass Cultures

**DOI:** 10.3390/ijms10010037

**Published:** 2008-12-27

**Authors:** Iwona Wilk–Zasadna, Maria Minta

**Affiliations:** Department of Pharmacology & Toxicology, National Veterinary Research Institute, Al. Partyzantow 57, 24–100 Pulawy, Poland. E-Mail: iwonawilk@o2.pl

**Keywords:** Ochratoxin A, developmental neurotoxicity, *in vitro* micromass cultures, embryonic midbrain cells, computer image analysis, immunocytochemistry

## Abstract

Embryonic midbrain micromass cultures were exposed for five days to ochratoxin A (OTA) at seven concentrations (ranging from 0.16 to 10 μg/mL). Cell viability was assessed in neutral red uptake test (NRU), and differentiation – by immunoenzymatic determination of structural proteins (β_III_-tubulin, MAP2, GFAP) expression level as well as by computer image analysis. Dose dependent decrease in cell number and differentiation was observed. Concentration-response curves were analysed and the mean inhibition concentrations (μg/mL) for cytotoxicity (IC_50_) and differentiation (ID_50_) were calculated. There were no significant differences in the sensitivity of neurons in early and late stage of differentiation and astrocytes to the toxic activity of this compound. For all endpoints ID_50_ value was very low (< 10 μg/mL) so OTA was classified as a strong teratogen. IC_50_/ ID_50_ ratios <2 pointed out that with harmful action of OTA the basic cytotoxicity should be connected.

## 1. Introduction

The developing central nervous system (CNS) is much more vulnerable to injury from toxic agents than the adult CNS. In fact, if we consider congenital defects, we see the brain as the major target of toxicity [1]. Moreover, CNS effects are seen most often at lower doses than those required to affect other parts of the body. Because of growing recognition of apparent increase in the incidence of developmental disabilities, considerable attention is focused on the effects of exposures to environmental pollutants such as methylmercury, lead, arsenic, polychlorinated biphenyls (PCBs), dioxins, and pesticides [2, 3]. Studies of food contaminants are of great importance [4].

Ochratoxin A (OTA) is a mycotoxin that occurs in improperly stored food products and is produced by some *Aspergillus* and *Penicillium* species. It primarily disrupts renal function and has been discussed as a causative factor in the human disease “Balkan endemic nephropathy” (BEN) and in the development of urinary tract tumours in humans [5]. OTA is also known to cause a wide range of other toxic effects, including carcinogenicity, hepatotoxicity, neurotoxicity, and teratogenicity [6, 7] as well as immunomodulating and often immunosuppressive action [8, 9].

Most of the available data on teratogenicity (developmental toxicity) of OTA comes from rodent studies in which developmental neurotoxicity was indicated by both morphological or behavioural changes. Pregnant golden hamsters injected i.p. once with 2.5–20 mg OTA/kg b.w. between 7–10 days of gestation increased prenatal mortality and on day 9 diminished foetal growth and malformations such as micrognathia, hydrocephaly, short tail, oligodactyly, syndactyly, cleft lip, micromelia, and heart defects occurred [10]. Embryocidal, foetotoxic and teratogenic effects of OTA were found in rats after a single s.c. dose of 1.75 mg/kg between days 4–10 of gestation [11, 12]. OTA at different graded dose levels (2–4 mg/kg body weight) and different gestation days (6–15), caused variable developmental defects (external hydrocephaly, incomplete closure of skull, and omphalocele) in foetuses [13]. A single oral dose of 2.75 mg/kg b.w. was found as the minimum effective teratogenic dose. Craniofacial malformations included exencephaly, midfacial clefting, and cleft lip were found in mice exposed to OTA [14]. Mice pups exposed prenatally to OTA were examined in surface righting (days 3–12), swimming, and pivoting tests [15]. Statistically significant differences for all three tests indicated that developmental delay had occurred; however no treatment or dose–related pathoanatomic alterations were found. The examination of brain regions of three month mice offspring exposed prenatally to OTA revealed that both, tissue weight and DNA content were reduced to 80% of control in cerebral hemispheres and 90% in remainder of the brain [16]. It was found that microcephalic brains in mice exposed to OTA were resulted from a reduced dendritic growth [17]. The treatment of postimplantation rat embryos in culture resulted in both concentration - dependent reduction in yolk sac diameter, crown-rump length, somite number count, and protein and DNA content as well as in an increase in the incidence of defective embryos (hypoplasia of telencephalon) [18].

Up to the present, the mechanism of toxic action of OTA on the prenatal development of the central nervous system has not been fully characterised. New *in vitro* strategies based on cell cultures are promising; however, the complexity of the nervous system requires the use of various cellular models representing specific *in vivo* targets [19 – 22]. The aim of this study was to gain more insight into the influence of OTA on the embryonic neural cells cultured in high density “micromass cultures”.

## 2. Materials and Methods

### 2.1. Animals — donors of embryonic midbrain cells

This study was carried out according to compliance with bioethical principles. Wistar albino rats were housed under standard laboratory condition of lighting (12 h dark/12 h light), temperature (20 ± 2 °C), and relative humidity (50 – 60%) with free access to commercial feed (Murigan) and tap water. Animals (10–12 week old) were mated overnight and the appearance of a vaginal plug on the next morning was considered as day one of pregnancy. Rat embryos were collected on day 13 of gestation. Procedures were followed essentially as described in *INVITTOX* Protocol No. 114 [23].

Tissues were pooled from a number of embryos, washed and incubated for 20 min at 37 ºC in sequence in Earle’s balanced salt solution calcium and magnesium-free (CMF, GIBCO), and 1% trypsin (GIBCO) in CMF. Then the tissues were washed in CMF and culture medium (Ham’s F-12 nutrient mixture: foetal bovine serum: l-glutamine: penicillin/streptomycin - 88:10:1:1 v/v – all materials were from GIBCO). A single cell suspension was ensured by mechanical dissociation followed by passing the suspension through sterile 10 μm nylon mesh. Viable cells density was assessed by trypan blue exclusion assay using Bürker’s haemocytometer.

### 2.2. Compounds tested

An ochratoxin A (OTA) analytical standard was purchased from SIGMA. Stock solutions of this compound were prepared by dissolving it in ethanol, whereas working dilutions (0.16 – 10.0 μg/mL) were prepared in culture medium. Penicillin G sodium salt (Pen-G, SIGMA) served as negative control and 5-fluorouracil (5-FU, SIGMA) as the positive one. Control plates included 5-FU at concentrations ranged from 0.015 to 1.0 μg/mL and Pen-G at the concentration of 500 μg/mL. Working dilutions of both control compounds were prepared in medium.

### 2.3. Embryonic nerve cell cultures

High-cell-density cultures were set up carefully as a 5–10 μL drop in the centre of each well of 96-well fibronectin coated microplate (BECTON DICKINSON), so that number of cells /well was 5 × 10^4^. After 2 h incubation (37°C, 5% CO_2_) the cells were exposed to different concentrations of tested compounds and incubated five days. Then dual assessment *i.e.* cytotoxicity and an impact of tested compound on cells differentiation (embryotoxicity) were done in the same cultures.

### 2.4. Cytotoxicity assay

Cytotoxicity was quantified using neutral red uptake test (NRU). The cells were fixed with 4.5% glutaraldehyd solution (SIGMA) for 30 min at room temperature and then washed with 0.9% phosphate buffered saline (PBS, SIGMA). After 30 min (room temperature) staining with 0.05% neutral red (SIGMA), acid alcohol (1% acetic acid in 50% ethanol) was added to extract neutral red from living cells. The eluted stain intensity (optical density, OD) was measured at 540 nm following at least 2 h incubation. Surviving cell number was directly related to the absorbance of the eluted stain.

### 2.5. Assessment of differentiation

#### 2.5.1. Computerised image analysis

After removing the eluted solution, the cells were fixed in 10% formaldehyde for 20 min and stained (approximately 10 s) with Gill’s haematoxylin. Morphometric analysis was performed on digitised images of cells taken with Nikon SMZ stereoscopic microscope linked to Panasonic CCD BP-KR 222E colour camera. The number of neurospheres (containing differentiated neural cells) was counted and their total and individual area was measured using computer image analysis software (LUCIA Measurement System).

#### 2.5.2. Immunostaining

To indicate the target cells damaged, immunofluorescent (IF) and immunoenzymatic (IE) staining was performed. Cells were fixed with 80% ice-cold acetone for 30 min in 4°C. To inhibit non-specific binding of immunoglobulin, the cultures were incubated in blocking buffer (10% FBS and 1% bovine serum albumin (BSA, SERVA) in PBS) at room temperature for 1 h. After rinsing with wash buffer (0.05% Tween-20 in PBS), three monoclonal antibodies (MA) were chosen and applied as primary antibody: MA anti- β_III_-tubulin isoform (Chemicon, 1:500 dilution), MA anti-microtubule associated protein 2 (MAP2) (Chemicon, 1:500 dilution), and MA anti-glial fibrillary acidic protein (GFAP) (Chemicon, 1:800 dilution). The primary antibody was applied overnight at 4°C at the given dilution in blocking buffer (50 μL/well). Then, after rinsing with wash buffer, the biotin conjugated anti-mouse secondary antibody (Chemicon, 1:200 dilution) was applied for 2 h (room temperature, 50 μL/well). The cells were then rinsed as above and incubated with streptavidin - FITC (Chemicon) or strepatividin - horseradish peroxidase (Chemicon) conjugate (50 μL/well) for 1 h at room temperature. IF staining fluorescence was examined using a fluorescent microscope (λ = 490 nm). In IE staining, a substrate (3,3’,5,5’-tetramethylbenzidine, SIGMA) was added (100 μL/well) and reaction was evaluated for 30 min at room temperature. By adding of 100 ∝ Λ 0.5 M sulfuric acid per well the reaction was stopped. The expression level of particular proteins was determined by measuring optical density at 450 nm.

### 2.6. Statistical analysis

The values of drug concentration, which inhibited cells viability by 50% (IC_50_) and reduced differentiation by 50% (ID_50_) when compared to unexposed control cells, were calculated according to Hill’s equation (sigmoidal model of concentration-response curve). Statistically significant differences between mean values obtained in immunoenzymatic analysis were determined based on analysis of variance (ANOVA) followed by Tukey – Kramer post-hoc test. Values of P < 0.05 were considered as statistically significant. All calculations were done using LSW Data Analysis Toolbox ver. 1.1.1. (MDL® Information Systems, Inc.) in Microsoft Excel 2000 spreadsheet and Kyplot software (Koichi Yoshioka, version 2.0 beta 4). In order to classify embryotoxic potential the mean inhibition concentrations for differentiation (ID_50_) of test chemical was used and the biostatistically based prediction model (PM) was applied [24]. It is based on the linear discriminate analysis of three functions: function I: 6.65 × log (ID_50_)–9.49; function II: 6.16 × log (ID_50_)–8.29; function III: –1.31 × log (ID_50_)–1.42. The function providing the highest value was used to determine the class of embryotoxicity (class 1 – non-embryotoxic, class 2 – weakly embryotoxic, class 3 – strongly embryotoxic).

## 3. Results

**Figure 1.**
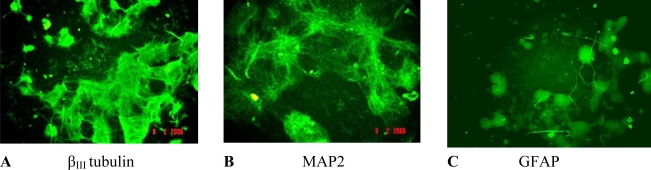
Photomicrographs showing control neurospheres stained using FITC- conjugated antibody against β_III_ tubulin, MAP2 and GFAP.

To ensure that our data meet all the acceptance criteria concomitant control cultures were carried out. We have ascertained that the medium culture values concerning cells viability and differentiation in this study corresponded well with the historical (cumulative) data obtained during in-house validation study of this method [25]. Immunofluorescent analysis revealed that control neurospheres (Figure 1) contain neurons in early (A) and late (B) stage of development as well as astrocytes (C).

The intensity of immunostaining was as follow: β_III_-tubulin> MAP2>GFAP. Immunoenzymatic analysis, showed that the most numerous were neurons in early stage of development (Table 1). Then, the model used was checked by assessment of culture response to reference compounds recommended by the European Centre for Validation of Alternative Methods (ECVAM) - 5-fluorouracil (5-FU) and penicillin G (Pen-G). Both, 5-FU and Pen-G were classified to the same classes (respectively class 3 – strong teratogens and class 1 – non-teratogens) as with use of the scientifically validated tests [24]. Pen-G was not embryotoxic at the concentration of 500 μg/mL. The mean values of cytotoxic (IC_50_) and embryotoxic (ID_50_) concentrations (± standard deviation, SD) for 5-FU are displayed in Table 1.

**Figure 2.**
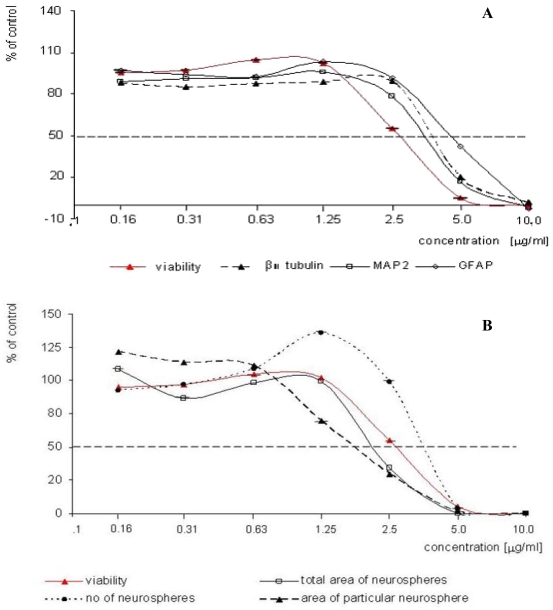
Representative concentration-response curves illustrating an effect of ochratoxin A on cytotoxicity (NRU test) and differentiation (six different endpoints) in micromass embryo midbrain cells cultured *in vitro*. The cytotoxic concentration of 2.5 μg/mL, mentioned in Table 1, resulted in increasing the number of neurospheres and decreasing of their area (B).

Cultures exposed to ochratoxin A showed dose–dependent reduction in total living cells number and differentiation (Figure 2). The mean cytotoxic concentration (IC_50_) calculated from three independent experiments was 2.52 ± 0.062 μg/mL. The mean inhibition concentration for differentiation (ID_50_) was also very low for all endpoints measured (Table 1). When prediction model was applied, OTA was classified as a strong teratogen (class 3). Small differences observed were depended on the criteria of evaluation.

Immunoenzymatic assessment of characteristic protein expression level did not show any significant differences in the sensitivity of particular embryonic nerve cells to toxic activity of OTA. The values of ID_50_ were 3.90 ± 0.350 μg/mL, 4.84 ± 0.581 μg/mL and 4.87 ± 0.351 μg/mL for MAP2, β_III_ tubulin and GFAP, respectively. IC_50_/ID_50_ ratio was similar for all three endpoints, *i.e.* 0.5 for β_III_ tubulin and GFAP, 0.6 for MAP2 (Table 1).

**Figure 3.**
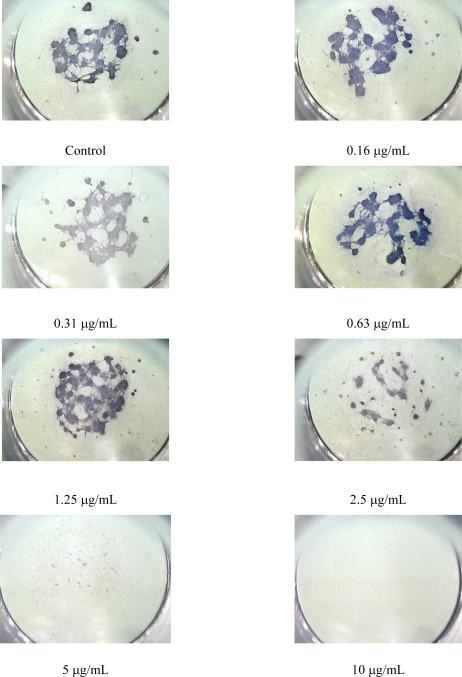
Images of midbrain micromass cultures, exposed for 5 days to different concentrations of OTA. Neurospheres were stained with haematoxylin (16 × magnification). After exposure to 2.5 μg/mL neurospheres were smaller and more numerous, after two highest concentrations no differentiated cells were observed.

Morphometric analysis of haematoxylin stained cultures revealed that the concentrations of OTA ranged from 0.63 to 2.5 μg/mL led to the decrease in the particular area and increase in the number of neurospheres (Figures 2B, Figure 3). This is probably reflecting a decreased intercellular adhesion Concentration response curve for total area was very similar to the illustrating the cytotoxicity (Figure 2B), so the IC_50_/ID_50_ ratio was 1.1 (Table 1).

## 4. Discussion

Nowadays, there is a trend towards toxicity testing with the use of short test systems *in vitro*. As concerning developmental neurotoxicity (DNT) different alternatives are used [19 – 22]. One of the neural cell-based model is the micromass test. The use of micromass cultures of cells derived from rat embryonic limb buds and midbrains has been developed and extensively characterised in the laboratory of Flint [26, 27], and then applied as *in vitro* teratogen assay [23, 28 – 30].

When cells isolated from midbrain of developing embryos are cultured at high density, many of the events (movement, communication, division and differentiation), which are most critical to embryogenesis, occur so this model, at least in part, resembles *in vivo* development. In principle, the micromass assay is based on detecting the ability of a particular chemical to inhibit the differentiation; two endpoint values are usually determined: ID_50_ for cell differentiation and IC_50_ for cell viability/growth.

Various approaches have been suggested to identify and characterise developmental toxicity from micromass assay. Most often teratogenic potential of a compound was based on the comparison of IC_50_ for cell differentiation and cytotoxicity [26, 31, 32]. According to this criterion, the compound is assumed as potentially teratogenic if it inhibits the differentiation at non-cytotoxic concentrations (IC_50_/ ID_50_ ratio>2). The functioning opinion is that the best results (concordance with *in vivo* studies and/or observations on human) are received when combination of two values (high IC_50_-P/ IC_50_-D ratio and very low IC_50_-D) is applied. Recently, Walmod *et al*. [33] hypothesised that a compound is likely to be a teratogen if it affects the proliferation or cell morphology, and that an effect on both endpoints, rather than just one of them, increases this probability. According to the authors, the concept of combining several endpoints in a single evaluation is well known and used.

In this study, OTA inhibited both viability and differentiation in a concentration – dependent manner. The mean inhibition concentrations of cell differentiation were closed to cytotoxic one (IC_50_/ID_50_ ratio <2). These findings are similar to those reported by Hong *et al.* [34] in rat embryonic limb bud and midbrain micromass cultures, and by Sava *et al.* [35] in adult hipocampal neural stem/progenitor cells *in vitro.* These results allow to conclude that harmful effects of OTA are related to the overt cytotoxicity [7, 36]. On the other hand it is known that cytotoxicity and specific toxicities are both underlying time kinetics so it is likely that harmful effects on differentiation could be see at an earlier timepoint during incubation.

Analysis of other *in vitro* data confirmed that cytotoxic potency of ochratoxin A is very high in different culture systems indicating that ochratoxin A did not have cell specific effects [37]. OTA has been suggested by various researchers to mediate its toxic effects *via* induction of apoptosis, disruption of mitochondrial respiration, inhibition of protein synthesis, oxidative stress, and generation of DNA adducts [38–41]. From our study a decreased intercellular adhesion was concluded resulting in inhibitory effects on aggregate formation.

As the development of the CNS is very complex, much effort should be done to improve *in vitro* cultures and to make the assessment of differentiation more objective [42, 43]. Embryonal CNS cells can proliferate in culture, generating aggregates called “neurospheres”. Cells in neurospheres express protein, among others β_III_ tubulin, MAP2, and GFAP, which were used as markers of neurons at early and late stage of development and astrocytes, respectively.

One possible mechanism of neuroteratogenicity is a target toxicity. Belmadani *et al.* [44] described cytotoxic potential of OTA (IC_50_) for protein and DNA synthesis in the culture of cells isolated from different part of rat embryo brain aged of 15–16 days. Median inhibition concentrations for neurons and astrocytes ventral mesencephalon were approximately 14 ± 2 μM (5.6 ± 0.40 μg/mL) and 40 ± 5 μM (16.2 ± 2.15 μg/mL) compared to the cerebellum values 24 ± 7 μM (9.7 ± 2.8 μg/mL ) and 69 ± 9 μM (27.9 ± 3.6 μg/mL). The same authors, in *in vivo* studies, designed the ventral mesencephalon, hippocampus, striatum, and cerebellum as the main OTA-targets in the brain of adult rats [45]. The regional selectivity of OTA was investigated in primary cultures of neurons and astrocytes isolated from embryonic or newborn rat brain ventral mesencephalon and cerebellum. The cultures were exposed to ochratoxin A in a medium containing 10% of foetal calf serum for 46 h. Neuronal cells were more sensitive than astrocytes, and the cells of the ventral mesencephalon were more sensitive than those of the cerebellum [46]. Zurich *et al.* [47] found out that OTA-induced alterations were more pronounced in cultures at an advanced stage of maturation because OTA may severely affect the neuroprotective capacity of glial cells (inhibition of glial fibrillary acidic protein). It seems to be that the differences mentioned above resulted from different experimental conditions, which may suggest that OTA toxicity is age and brain region depended. The risk for human health depends on many factors.

## 5. Conclusions

Overall, our results demonstrates that OTA is a strong teratogen (ID_50_ < 10 μg/mL) and its primary harmful effect is general cytotoxicity (similar IC_50_ and ID_50_). Immunoenzymatic assessment did not show selective toxicity of OTA to particular neurons and astrocytes. Morphometric assessment using computer image analysis revealed that teratogenic potency of OTA was correlated with their effects on aggregation of neuronal cells and formation of neurospheres. Taken together, the use of different endpoints for evaluation of differentiation may offer an interesting improvement of the CNS micromas test which might be used, also, for mechanistic studies.

## Figures and Tables

**Table 1.** Effects of 5-fluorouracil (5-FU) and ochratoxin A (OTA) on viability (NRU test) and differentiation (based on six endpoints).

Endpoint	Viability	Differentiation					
	Neutral Red OD, 540 nm	Computerised image analysis of haematoxylin stained neurospheres			Expression of proteins (OD, 450 nm)		
		Number	Total area mm^2^	Particular area mm^2^	β_III_ tubulin	MAP2	GFAP
Controls mean±SD, n- number of wells	1.454±0.2084 n =232	61±36 n = 435	2.56±1.13 n = 435	0.112±0.059 n = 484	2.592±0.529* n = 141	1.923±0.440* n = 207	1.689±0.405* n = 119
	Cytotoxicity IC_50_	Inhibition of differentiation, ID_50_					
5-FU**	0.15±0.010	0.17±0.016	0.16±0.010	0.21±0.015	0.14±0.010	0.16±0.010	0.18±0.012
IC_50_/ID_50_ ratio		0.9	0.9	0.7	1.2	0.9	0.8
OTA**	2.52±0.062	3.40±1.100	2.19±0.245	1.29±0.923	4.84±0.581	3.90±0.350	4.87±0.351
IC_50_/ ID_50_ ratio		0.7	1.1	1.9	0.5	0.6	0.5

* P<0.05, significant difference was calculated for each pair of means in Tukey–Kramer test

** μg/mL, mean±SD - from 3 independent experiments
